# The Influence of Web-Based Tools on Maternal and Neonatal Outcomes in Pregnant Adolescents or Adolescent Mothers: Mixed Methods Systematic Review

**DOI:** 10.2196/26786

**Published:** 2021-08-26

**Authors:** Jania J Y Wu, Nurulhuda Ahmad, Miny Samuel, Susan Logan, Citra N Z Mattar

**Affiliations:** 1 Yong Loo Lin School of Medicine National University of Singapore Singapore Singapore; 2 Department of Obstetrics and Gynaecology National University of Singapore Singapore Singapore; 3 Research Support Unit Yong Loo Lin School of Medicine National University of Singapore Singapore Singapore; 4 Department of Obstetrics and Gynaecology Yong Loo Lin School of Medicine National University of Singapore Singapore Singapore

**Keywords:** pregnancy in adolescence, teenagers, adolescents, pregnancy, postpartum, internet, digital health, digital media, new digital media, eHealth, social media, social network, communications media

## Abstract

**Background:**

Pregnant adolescent women increasingly seek support during pregnancy and the puerperium through digital platforms instead of the traditional support system of family, friends, and the community. However, it is uncertain whether digital, web-based tools are reliable and effective in providing information to the user on a variety of topics such as fetal development, pregnancy outcomes, delivery, and breastfeeding to improve maternal and infant outcomes.

**Objective:**

We aimed to identify web-based tools designed to promote knowledge, attitudes, and skills of pregnant adolescents or adolescent mothers and determine the efficacy of such web-based tools compared with conventional resources in promoting good pregnancy and infant outcomes.

**Methods:**

A systematic search was conducted using Medline, Scopus, CINAHL, and PsycINFO for articles published from January 2004 to November 2020 to identify randomized trials and observational studies that evaluated digital, web-based platforms to deliver resources to pregnant adolescents. All articles written in the author’s languages were included. Two authors independently reviewed abstracts and full-text articles for inclusion and assessed study quality. Risk of bias in each study was assessed using appropriate tools recommended by PRISMA (Preferred Reporting Items for Systematic Reviews and Meta-analyses) and the Joanna Briggs Institute. We adopted a qualitative synthesis and presented the results in a narrative format due to the heterogenous nature of the studies.

**Results:**

Seven articles met the inclusion criteria and were analyzed. The majority of the studies were graded to be of low to moderate risk for bias. The research methodologies represented were varied, ranging from randomized (n=1) and nonrandomized controlled trials (n=1) and prospective cohort studies (n=1) to mixed methods studies (n=1) and longitudinal surveys (n=3). Four studies included active web-based interventions, and 3 described exposure to web-based tools, including the use of social media and/or other internet content. Web-based tools positively influenced treatment-seeking intentions (intervention 17.1%, control 11.5%, *P*=.003) and actual treatment-seeking behavior for depression among postpartum adolescents (intervention 14.1%, control 6.5%, *P*<.001). In contrast, readily available information on the internet may leave adolescents with increased anxiety. The critical difference lies in information curated by health care professionals specifically to address targeted concerns versus self-acquired data sourced from various websites.

**Conclusions:**

Despite almost universal web use, few studies have used this platform for health promotion and disease prevention. Social media interventions or web-based tools have the potential to positively influence both maternal and infant outcomes in adolescent pregnancy, but there is a need for more well-conducted studies to demonstrate the effectiveness of these support programs. The vastness of the information available on the web limits the ability of health care professionals to monitor or control sources of information sought by patients. Thus, it is important to create professionally curated platforms to prevent or limit exposure to potentially misleading or harmful information on the internet while imparting useful knowledge to the user.

**Trial Registration:**

PROSPERO International Prospective Register of Systematic Reviews CRD42020195854; https://www.crd.york.ac.uk/prospero/display_record.php?RecordID=195854

## Introduction

Pregnant adolescents are an especially vulnerable population. Despite the significant decline of adolescent pregnancies in recent decades [1], the World Health Organization estimates that 12 million girls aged 15 to 19 years give birth yearly in developing countries [2]. These adolescents are at increased risk of prenatal and perinatal complications including gestational hypertension, preterm delivery, low infant birth weight, and other neonatal complications [3-5]. Adolescent pregnancies are more prevalent in socioeconomically disadvantaged communities, on a background of disrupted family structures and limited educational opportunities [6]. Poor pregnancy outcomes are more frequent among socioeconomically disadvantaged adolescents, largely due to the complex social and cultural factors that result in lower or delayed maternal engagement with health care services [7,8].

The transition from child-free adolescence to motherhood is daunting. Traditionally, those who are pregnant or postpartum turn to their family, friends, and partners for support [9]. More recently, community-based and home-visit programs have also been established to support adolescent mothers. Home-visit programs may offer better outcomes in adolescents who are difficult to engage due to close bonds formed [10]. These programs aim to provide access to information, resources, and social support in order to maximize coping strategies and eventual reintegration into society [11,12]. The Teenage Pregnancy Strategy is an example of a successful, multicomponent intervention that has reduced adolescent conceptions and improved outcomes for adolescent mothers by providing support for mothers targeted at completion of education and securing appropriate housing [13].

With increasing access to technology, expectant mothers may seek pregnancy-related information or support from social media [14,15] or internet-based platforms [16,17] instead of traditional sources. Due to shifts in contemporary social structure, many women find themselves geographically and emotionally isolated from their support system of family and friends [14,15]. Alternative support systems on digital platforms provide opportunities for like-minded women to exchange experiences and gain social support. Web-based support systems are available regardless of time and location and allow for anonymity of use, reducing stigma and facilitating the discussion of sensitive topics [18]. Furthermore, the majority of expectant mothers perceive the internet to be a reliable source of information and access information on a large variety of topics including fetal development, delivery, and breastfeeding [16]. As such, information found on the internet has the potential to influence the mother’s decisions surrounding her pregnancy care [19].

While the definition of social media is dynamic and constantly evolving [20], in general, a social media site is an interactive online platform that facilitates the exchange of user-generated content [21,22]. In our study, we defined social media as any online platform that enables users to exchange content (eg, Facebook or Instagram), while internet content was defined as any online platform that does not facilitate content exchange among users (eg, websites or online reading materials) [21,22]. We defined web-based tools broadly as describing all services and technologies found on online platforms and consider both social media and internet content to be subsets of web-based tools [23]. Despite their convenience and easy access [24], these tools have limitations. Information found online may not be verified and may provide pregnant women with inaccurate, unreliable, or unsupported knowledge [25]. A meta-analysis evaluating the quality of online health information found that 70% of the included studies concluded that information sources on the internet were of low quality [26] and often provided advice with limited or no scientific evidence [27-29]. Specifically regarding women’s health, inaccurate celebrity-based advice has been highlighted [30]. Low-quality pregnancy-related information may be harmful or conflicting [29] and is often not discussed with health care providers to clarify misconceptions [24,31], all of which have the potential to negatively influence pregnancy outcomes. The unregulated online community can also produce negative experiences for the naïve user [32]. As the use of social media or internet resources during pregnancy is a relatively recent phenomenon, there is an opportunity to explore the association between its use among at-risk adolescents and perinatal outcomes.

The aims of this systematic review were to assess the impact of web-based tools used by pregnant adolescents or adolescent mothers on maternal and infant outcomes to compare these to conventional resources and critically appraise the evidence from relevant quantitative and qualitative studies. The research questions addressed were as follows:

- What types of available web-based tools are designed to promote knowledge, attitudes, and skills of pregnant adolescents or adolescent mothers?
- How effective are these web-based tools in promoting good pregnancy and infant outcomes compared with conventional resources?

## Methods

### Search Strategy

The protocol was registered with the International Prospective Register of Systematic Reviews (PROSPERO) [CRD42020195854]. We followed the Preferred Reporting Items for Systematic Reviews and Meta-analyses (PRISMA) guidelines [33] and conducted a systematic search of PubMed, Scopus, CINAHL, and PsycINFO electronic databases for articles published from January 2008 to November 2020. We restricted publications to the last 12 years to ensure studies promoted updated practices relating to pregnancy care. The search was initially conducted on May 15, 2020, and updated on December 5, 2020. The process of updating the search was guided by methods described by Bramer et al [34]. We also conducted a secondary search to identify studies published between 2004 and 2007 as the first concept of Web 2.0, which is defined as a network platform that spans across all devices, was introduced in 2004 [35]. No further studies met our inclusion criteria. Two librarians from the National University of Singapore Medical Library were consulted on the finalization of the search strategy. Search terms included “pregnant,” “adolescents,” “social media,” and “internet,” and the full search strategy can be found in Multimedia Appendix 1.

### Selection of Studies

The study selection was conducted in two phases. During level 1 screening, two authors (JW, NA) independently screened all studies retrieved by electronic database searches based on key terms and resolved discrepancies by discussion with a third author until a list of studies for level 2 screening was agreed upon. During level 2 screening, the full texts of studies selected in level 1 were retrieved and independently reviewed by the same 2 authors to determine the eligibility of each study; discrepancies were again resolved by discussion with the third author until a final list of studies was agreed on. The reasons for exclusion were coded and recorded systematically.

### Eligibility Criteria

All inclusion and exclusion criteria were defined a priori. We included both quantitative and qualitative studies that explored the association between the use of web-based tools by pregnant adolescents or adolescent mothers and maternal and neonatal outcomes. Studies that defined their population as adolescents or studied women aged 21 years and younger were included. Social media was defined as online platforms providing avenues to exchange content with other users (eg, Facebook, Instagram, Twitter, blogs, vlogs, forums, chatrooms) while internet content was defined as online platforms that did not provide direct opportunities to interact with other users (eg, websites, online reading materials, internet programs). Conventional tools developed to support adolescent mothers such as brochures, school-based counseling, community-based counseling, and group and personal counseling were the comparison of interest. Maternal outcomes measured were physical (nutrition, physical activity, breastfeeding practices, birth complications, and risky behaviors such as smoking and alcohol consumption) and psychosocial (mental health, depression, anxiety, loneliness and stress, self-esteem, birth preparedness, and parenting outcomes) factors. Infant or child outcomes included preterm birth, low birth weight, sudden infant death syndrome, and obesity. All outcomes collected met the inclusion criteria. We included studies with or without a comparison group that were relevant to answering our research questions and excluded reviews, abstracts, conference proceedings, letters, editorials, comments, opinions, and book chapters. We excluded studies that were not in English, Chinese, Malay, or French, the languages of the authors. Studies that only examined the use of social media or internet content with no quantitative or qualitative outcomes were also excluded.

### Data Extraction

We extracted relevant evidence using a standard proforma including study design, settings, observational period, sample size, participant characteristics, description of intervention and comparison tools, maternal outcomes, infant or child outcomes, adjusted factors, findings, and limitations.

### Risk of Bias Assessment

We conducted a mixed methods systematic review to assess the relevant studies using various critical appraisal tools that are validated and widely used. Qualitative studies [36] and nonrandomized controlled trials [37] were evaluated using the Joanna Briggs Institute instrument, which appraises articles as “included,” “excluded,” or “seek further info” based on 10- and 9-question checklists, respectively. Cohort or case-control studies were evaluated using the Newcastle-Ottawa Quality Assessment Scale [38], which rates articles according to selection, comparability, and exposure categories using a star system. Randomized controlled trials (RCT) were evaluated using the Cochrane Risk of Bias 2 describing a “fixed set of domains of bias, focusing on different aspects of trial design, conduct, and reporting” [39]. It appraises articles as low risk of bias, some concerns, and high risk of bias. Studies using mixed methodologies were evaluated using the Mixed Methods Appraisal Tool [40] based on a qualitative and quantitative scoring system. Four authors (JW, NA, SL, CM) independently and critically appraised each study for quality and potential bias. We resolved discrepancies by discussion until a consensus was reached.

### Synthesis of Results

We categorized included studies into 2 groups to answer our research questions. To explore the variety and effectiveness of web-based tools in promoting knowledge, we analyzed and compared all 7 studies [41-47]. To determine their influence on pregnancy outcomes compared to conventional resources, we assessed 2 studies [41,42]. The studies analyzed were too heterogeneous to enable a formal meta-analysis, thus we adopted a qualitative synthesis and presented the results in a narrative format.

## Results

### Search Results

**Figure 1:**
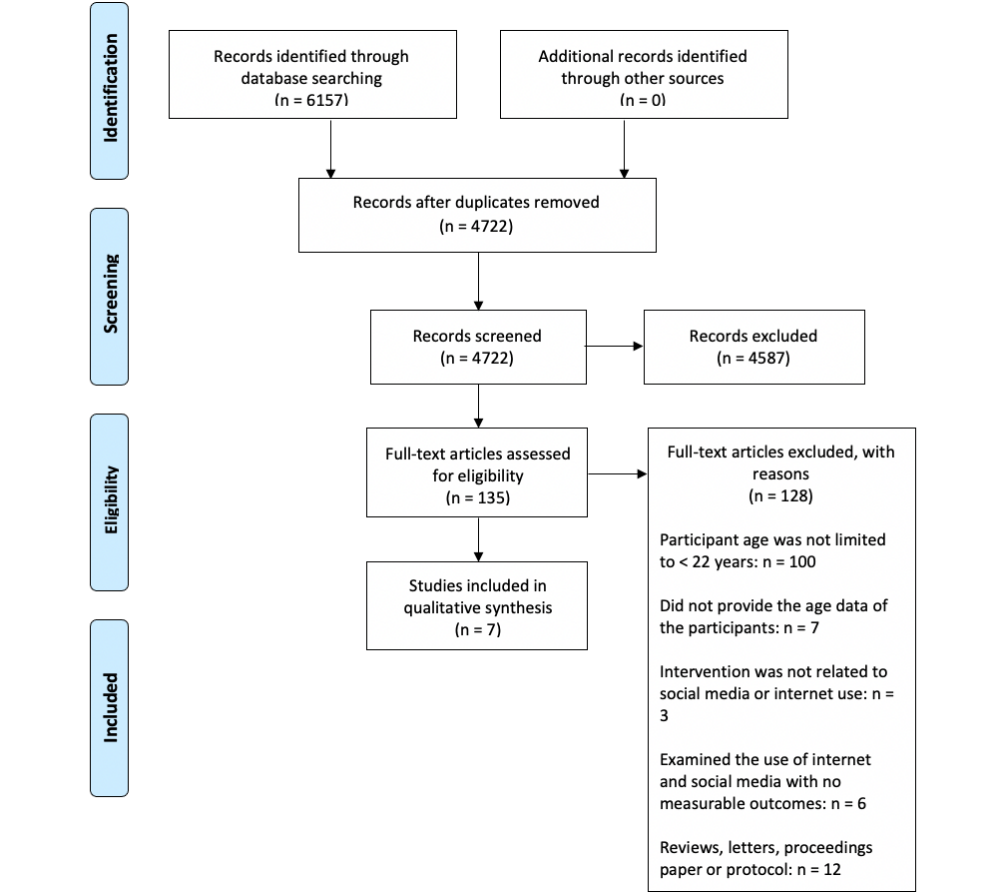
PRISMA flow diagram.

The search in the various medical databases (Medline [using PubMed platform], Scopus, CINAHL [using EbscoHost platform], and PsycINFO) for articles published from January 2008 to November 2020 yielded a total of 6157 records. After removing the duplicates, 4722 records were eligible for further screening. After 2 reviewers screened all titles and abstracts, 135 records met the inclusion criteria and were eligible for further full-text screening. Following the full text review, we excluded 128 articles based on the predefined exclusion criteria, and 7 articles were finally included in the systematic review for analysis (Figure 1).

### Study Characteristics

Characteristics of the 7 studies are summarized in Table 1. Six were conducted in the United States [41-44,46,47], and one was conducted in Western Australia [45]. The research methodologies represented were varied—the most common study design was qualitative (n=3) [43,45,46], while the remaining studies were an RCT [41], a non-RCT [47], a prospective cohort study [42], and a mixed methods study [44]. Study sizes ranged from 7 [43,45] to 292 participants [42]. Despite limiting our inclusion criteria to adolescents, the participant age range was large, from age 13 years [42] to a participant aged 22 years, who was included as she was considered an adolescent by the authors [42,46]. Most participants were defined as adolescents in 6 studies [41,42,44-47], while 1 study defined participants as first-time mothers [43]. Participants were currently pregnant (n=1) [44], already mothers (n=5) [41-43,45,47], or both (n=1) [46]. Various methods were used to encourage participation: 5 studies recruited participants from established institutions such as prenatal clinics or organizations offering services for adolescents [41,42,44,46,47], while the remaining 2 studies recruited participants by posting public advertisements or through personal and professional contacts [43,45]. Two studies included a control group, which received conventional care [41,42].

**Table 1:** Descriptive characteristics.

Author, country	Study design	Participants	Intervention/exposure	Control group (if any)	Outcomes evaluated	Key findings
Hudson et al [41], US	Quantitative; RCT^a^	Adolescents: 16-21 years (mean 18.3 [SD 1.7] years); 1-week postpartum; single, low-income, African Americans	n=15; NMN^b^ website: internet-based education resource, discussion forum, direct email contact with nurses (6 months)	n=19; usual care: hospital parenting instructions, parent’s own resources	Maternal: Mental health Parenting outcomes Birth complications Infant: Health care use Breastfeeding	The NMN website is well poised for nursing-driven social support intervention. The social support component was identified as a key strength with positive qualitative comments.
Logsdon et al [42], US	Quantitative; matched prospective cohort study	Adolescents: 13-21 years; up to 1-year postpartum; living in urban, suburban and rural counties, mixture of White (8.6%), Black (88.0%) and others (3.4%)	n=154 (mean 17.9 [SD 2.1] years); internet intervention website: internet-based education resources (2 weeks)	n=138 (mean 18.2 [SD 1.9] years); home visitation program	Maternal: Mental health	The internet intervention was successful in changing attitudes, perceived control, intention to seek treatment, and actually seeking treatment. The intervention effect was equal in adolescents regardless of where they lived, but the impact on changing attitudes may be dose dependent.
Fleming et al [43], US	Qualitative	First-time mothers; mean 18-21 [SD 19.5] years); 6-8 weeks postpartum; single (85.7%), low-income	n=7; personal electronic media use: websites, internet blogs, internet chat rooms, online shows (duration not specified)	—^c^	Maternal: Birth preparedness Mental health	This study demonstrated adolescents’ desire and need for clear, accurate, and easily accessible information about birthing. Providing credible electronic sources will educate the mothers and increase their confidence and birthing preparedness levels.
Vander Wyst et al [44], US	Mixed methods; non-RCT	Adolescents: 14-18 years; 12-28 weeks pregnant; low-income, mixture of Black (70%), Hispanic White (20%), and non-Hispanic White (10%)	n=10 (median 17.0 [IQR^d^ 16.4, 17.7] years); social media intervention: private Facebook group with interactive activities and dissemination of health information (18 weeks)	n=12 (median 29.2 [IQR 23.7, 33.8] years); adult participants^e^	Maternal Physical anthropometric data Nutrition knowledge Nutrition behavior Physical activity Attitudes and beliefs on prenatal health Social support Infant Birth weight Gestational age Breastfeeding	Poor diet quality persists among both adolescent and adult low-income pregnant women. Although social media-based education was well received by the participants, this did not result in significant changes in dietary intake and knowledge.
Nolan et al [45], WA^f^	Qualitative	Adolescents; 16-19 years; 3-17 months postpartum; single, living with parents (71.4%), extended relatives (14.3%), or partner/friend (14.3%)	n=7; personal social network site use: website that enables users to create public profiles and form relationships with other users (duration not specified)	—	Maternal: Social support Mental health Parenting outcomes	The use of social network sites affords adolescent mothers access to tangible, informational, and emotional support. There is a potential role for midwives to use such platforms to provide additional social support.
Rueda et al [46], US	Qualitative	Adolescents; 14-22 years; currently pregnant or mothers; single, living in residential foster care home, mixture of ethnic minorities: Hispanic (43.5%), Black (30.4%), Mixed race (10.9%)	n=13; personal electronic media use: social media websites, phone apps that facilitate communication between individuals (duration not specified)	—	Maternal: Relationship with intimate partners Mental health Infant: Child protection	The use of technology among adolescent mothers living in foster homes is associated with multiple social issues. Technology should be included in various models of care to increase understanding between professionals and adolescents.
Logsdon et al [47], US	Quantitative; non-RCT	Adolescents; mean 16.8 years; mothers; single, students of a public school–based program for adolescent parents; mixture of African American (48.6%), White (34.1%), and others (17.3%)	n=138; internet intervention website: internet-based education resources (single class period)	—	Maternal Mental health	The testing of a prototype website for adolescent mothers with postpartum depression showed promising results. Attitudes related to depression and seeking treatment improved after viewing the website.

^a^RCT: randomized controlled trial.

^b^NMN: New Mothers Network.

^c^Not applicable.

^d^IQR: interquartile range.

^e^Control group (adult participants) is not relevant to answering the research question.

^f^WA: Western Australia.

### Risk of Bias Within Studies

Table 2 illustrates an overview of the studies’ risk of bias. A detailed assessment of the relevant studies using various critical appraisal tools is found in Multimedia Appendix 2, as the criteria for each study differed by study design. All 7 studies were included, and 6 were judged to have a low risk of bias [41-46]. Of note, 2 out of 7 studies [42,47] were conducted by Logsdon et al [47]. The earlier study in 2013 was conducted as a pilot to test the prototype of a web-based intervention. Although included, it was rated as a poor-quality study due to the lack of a control group, the one-off nature of outcome sampling, lack of meaningful clinical outcome, and an inadequate description of statistical analysis used. This was subsequently followed by a more robust prospective cohort study in 2018 [42].

**Table 2:** Overview of the studies’ risk of bias.

Author	Study design	Quality assessment instrument	Rating
Hudson et al [41]	Quantitative; RCT^a^	Cochrane Risk of Bias 2	Include; risk of bias: low
Logsdon et al [42]	Quantitative; matched prospective cohort study	Newcastle-Ottawa Quality Assessment Scale (Cohort studies)	Include; selection: ***; comparability: **; outcome: **; risk of bias: low
Vander Wyst et al [44]	Mixed methods; non-RCT	MMAT^b^	Include; risk of bias: low
Logsdon et al [47]	Quantitative; non-RCT	JBI^c^ (quasi-experimental studies)	Include; risk of bias: moderate-high
Fleming et al [43]	Qualitative	JBI (qualitative research)	Include; risk of bias: low
Nolan et al [45]	Qualitative	JBI (qualitative research)	Include; risk of bias: low
Rueda et al [46]	Qualitative	JBI (qualitative research)	Include; risk of bias: low

^a^RCT: randomized controlled trial.

^b^MMAT: Mixed Methods Appraisal Tool.

^c^JBI: Joanna Briggs Institute.

### Synthesis of Results

The study results are summarized in Tables 3 and 4. Adolescents in 4 studies [41,42,44,47] received an active intervention. Of these studies, 1 included adolescent–health care professional interactions [41], 2 described adolescent-adolescent interactions [41,44], and the remaining 2 studies described purely internet content [42,47]. The adolescents in the remaining 3 studies [43,45,46] were exposed to various web-based tools. Of these studies, the duration of exposure to the web-based tools was not known for all [43,45,46], and resources accessed by the adolescents include informative [43] and social media websites [43,45,46]. Various outcomes were collected during and after the intervention, measured via self-reports, postintervention surveys, corroboration with medical records, validated tools (if available), and general anthropometric data. The outcomes are discussed as maternal and infant outcomes. We further categorized maternal outcomes into physical, mental well-being, parenting outcomes, and others.

**Table 3:** Synthesis of quantitative results.

Author, country	Statistically significant outcomes with intervention (*P*<.05, unless stated otherwise)	Non–statistically significant trends following intervention
Hudson et al [41], US	Assuming α=.10, *P*<.10; Intervention group had lower self-esteem than control group at 6 months; scale: RSE^a^ Intervention group had higher levels of perceived competence after 6 months; scale: PPS^b^ Intervention group had higher parenting satisfaction levels after 6 months; scale: WPBL-R^c^ ER^d^ use reduced >50% in intervention group compared to control group (35.7% vs 70.6%); data collection: questionnaire Intervention group was less likely to exclusively breastfeed compared to control group; data collection: questionnaire	Increasing: Social support No differences in: Depression symptoms Loneliness Perceived stress Birth complications
Logsdon et al [42], US	Intervention group had more positive attitudes toward seeking psychological help than the control group after 2 weeks; scale: ATSPH^e^ Intervention group had more positive perceived behavior control than the control group after 2 weeks; scale: HSDI^f^ Intervention group had greater intention to seek treatment for depression than the control group after 2 weeks; scale: MHI^g^ Intervention group had higher treatment seeking behavior for depression than the control group after 2 weeks; data collection: questionnaire	No differences in: Depression symptoms Stigma for receiving psychological help
Vander Wyst et al [44], US	There was higher sugar intake in both groups after 18 weeks compared to baseline; data collection: 24-hour diet recall calculated via FPP^h^ There was a lower likelihood of adolescents cooking at home at baseline compared to adults; data collection: questionnaire There was a lower likelihood of adolescents buying their own groceries at baseline and after 18 weeks compared to adults; data collection: questionnaire Adolescents were less knowledgeable in nutrition (eg, identifying fiber rich food, recommended whole grain consumption, fruit, vegetable and fat intake) compared to adults at baseline and/or after 18 weeks; data collection method: questionnaire	No differences in: Participant anthropometric data Mean caloric consumption Macronutrient distribution of food Infant birth weight Infant gestational age
Logsdon et al [47], US	Adolescents had more positive attitudes toward seeking psychological help postintervention compared to baseline; scale: ATSPH	No differences in: Mental health acceptability Stigma for receiving psychological help

^a^RSE: Rosenberg Self-Esteem.

^b^PPS: How I Deal With Problems Regarding Care of My Baby.

^c^WPBL-R: What Being the Parent of a Baby is Like–Revised.

^d^ER: emergency room.

^e^ATSPH: Attitude Toward Seeking Psychological Help.

^f^HSDI: Health Self Determination Index.

^g^MHI: Mental Health Intention.

^h^FPP: Food Processor Program.

**Table 4:** Synthesis of qualitative results.

Study, country	Outcomes
Fleming et al [43], US	Increased anxiety due to graphic media, birthing process, potential complications, and neonatal care Birth preparedness: suboptimal birth preparedness due to fragmented, inconsistent, weakly linked, and poorly referenced information although a small subset of women developed improved or enhanced understanding Social support: platform allowed connection with others and peer support
Vander Wyst et al [44], US	Nutrition behavior: adolescents had improved attitudes toward nutrition with dietary changes (eg, limiting high fat fast food, increasing vegetable and fruit intake), motivated by time, convenience, and food preferences Physical activity: adolescents had an increased tendency to exercise during pregnancy as they believed it to help with labor Breastfeeding: adolescents tended to be less likely to breastfeed compared to adults Social support: both adolescents and adults had both good and poor sources of social support
Nolan et al [45], WA^a^	Increased social support and connectedness: participants had unlimited access to relationships, minimizing feelings of exclusion, and social isolation. They could maintain both old and new friendships. Social network sites provide valuable tangible, emotional, and informational support for adolescent mothers, contributing to mothers’ social capital Parental stress and anxiety: social network sites served as a medium for problem sharing and helped to reduce parental stress and anxiety. Drawbacks were the absence of adequate privacy controls and negative comments that could potentially threaten emotional well-being Increased parenting confidence: peer support and positive affirmations significantly increased adolescents’ confidence levels
Rueda et al [46], US	Social media tools provided positive experiences in: Interacting with a potentially intimate partner Maintaining contact and fostering feelings of closeness with their child’s father Social media tools provided negative experiences in: Unwanted sexual solicitations Child protection, as meetings with strangers in offline spaces place both the adolescent and their children at risk Cyber abuse (eg, cyber bullying, stalking) of which adolescents were both victims and perpetuators Adverse emotional side effects fueled by jealousy and mistrust

^a^WA: Western Australia.

### Maternal Outcomes

Maternal mental well-being was explored in 5 studies [41-43,45,47]. Hudson et al [41] conducted an RCT comparing participants who were exposed to the New Mothers Network website with those who received typical parenting instructions provided by the hospital. The New Mothers Network intervention provided parenting information through their electronic library and via communications with other mothers and nurses. The authors found that self-esteem levels measured via the Rosenberg Self-Esteem scale were significantly lower in the intervention group over the 6-month period (*P*=.04), although the authors found no clear explanation for this trend. In the 2 studies conducted by Logsdon et al [42,47], the earlier prospective pilot study [47] explored the efficacy of a website showcasing pictures and stories of other adolescent mothers’ experiences, county and national mental health resources, and a frequently asked questions segment. The evidence from this study suggests that attitudes toward seeking psychological help, measured via the Attitude Toward Seeking Psychological Help scale, were significantly higher postintervention (*P*=.02). A similar result was observed in their prospective cohort study [42] of participants who received exposure to the website and home visits by volunteers as part of a home visitation program. Compared to the earlier pilot study [47], the intervention was adapted slightly to showcase video vignettes instead of pictures and stories based on user feedback. Mental health community resources and the frequently asked questions segment remained. The earlier results of improved attitudes toward seeking psychological help (*P*=.04) were successfully replicated, with additional data demonstrating that perceived behavior control (*P*=.007), intention to seek treatment for depression (*P*=.003), and seeking treatment for depression (*P*<.001) were significantly higher after 2 weeks in the intervention group compared to the controls. Data was measured using the Attitude Toward Seeking Psychological Help scale, Health Self Determination Index scale, Mental Health Intention scale, and a questionnaire on the outcomes of seeking treatment, respectively. Fleming et al [43] conducted qualitative interviews of participants who had prepared for childbirth through a variety of web-based tools including websites, blogs, chatrooms, and mass media. The findings suggested that access to electronic media did not necessarily prepare adolescents for pregnancy and childbirth but instead increased anxiety levels. Nolan et al [45] also conducted qualitative interviews on participants who used social media and reported that communicating with other adolescent mothers via social network sites helped reduce loneliness, parental stress, and anxiety.

Only one study by Vander Wyst et al [44] explored maternal physical outcomes. In this 18-week longitudinal study, participants were added into a private Facebook group where health information (pregnancy fitness, healthy recipes, nutrition, pregnancy fun facts, and stress management) were disseminated and interactive group activities were conducted. The authors compared adolescent and adult mothers and found dietary sugar distribution among both groups to be significantly increased after 18 weeks, with a greater increase in the adolescent group (7.4 [SD 0.2] vs 6.3 [SD 0.1] g/d, *P*=.005). Adolescent mothers were less likely to shop for groceries (postintervention; 14.0% vs 89.0%, *P*=.002) or cook at home (postintervention; 14.0% vs 67.0%, *P*=.054) compared to adult mothers and had significantly less knowledge regarding nutrition (identifying fiber rich foods, recommended daily consumption of wholegrains, fruit and vegetable varieties, and fat) before and after the intervention. There were no significant differences concerning anthropometric data, mean caloric consumption, or macronutrient distribution of food.

Parenting outcomes were explored in 3 studies [41,43,45]. Hudson et al [41] found that both intervention and control groups had significantly higher perceived competence (*P*<.01) and parenting satisfaction (*P*<.01) measured by the How I Deal With Problems Regarding Care of My Baby and evaluation subscale of the What Being the Parent of a Baby is Like–Revised, respectively. Fleming et al [43] concluded that although many young mothers had acquired knowledge on what to expect during childbirth, much of this was fragmented, inconsistent, weakly linked, poorly referenced, not always beneficial, and potentially a greater source of confusion. In contrast, Nolan et al [45] found that using social network sites increased young mothers’ confidence levels in parenting roles and with parenting strategies through positive affirmations, reassurance by other parents, and the collective sharing of experiences. Regarding social support to adolescent mothers, Hudson et al [41] observed a positive trend in social support levels following intervention supported by the qualitative comments submitted by participants. Nolan et al [45] described valuable tangible, emotional, and information support from social networks. Additionally, Rueda et al [46] explored the role of technology and social network sites on intimate relationships among adolescents living in residential foster homes. They determined these tools to be critical to the adolescent mothers’ ability to maintain or initiate intimate relationships with the fathers of their children or with new partners, facilitating both communication and in person meetings.

### Infant Outcomes

Infant outcomes were studied less. None of the studies investigated putative associations between the use of web-based tools and premature birth, low birth weight, infant obesity, or sudden infant death syndrome. Hudson et al [41] found that emergency services use for postpartum problems in the first 6 months decreased significantly following intervention compared to no intervention, as 70.6% of mothers who did not receive intervention brought their child to the emergency room at least once compared to 35.7% of mothers who received the intervention (*P*=.052). In each group, there was one visit to the emergency room that was appropriate, with one infant hospitalized and a mother-infant pair treated for smoke inhalation. Vander Wyst et al [44] described adolescents as less likely to breastfeed compared to adults. Hudson et al [41] observed that adolescents in the intervention group were less likely to exclusively breastfeed compared to those who received usual care (*P*=.06 [assuming α=.10, *P*<.10]). Rueda et al [46] reported that residential foster home staff were concerned about adolescent mothers bringing their children to meetings arranged over social media with unknown partners, potentially placing their children at risk. While this topic was not discussed with the adolescent mothers, their dialogue suggests that they are aware of such risks. This belies an important knowledge and perception gap of program staff on how adolescent mothers use technology and social networks, as meeting strangers offline was either not as common as program staff believed or was regarded as shameful and not openly discussed by the adolescents.

## Discussion

### Principal Findings

Based on current available literature, there is much that needs to be explored concerning the potential benefits and harms of social media for pregnant and postnatal adolescents. This is despite most adolescent participants having grown up in the presence of advanced technology and with extensive social media use. We have systematically studied the impact of web-based tools used by adolescents on various maternal and infant outcomes to address our research questions. We found that available web-based tools include professionally curated programs (via websites or social media), readily available information on the internet, and personal use of various social media platforms.

While the limited evidence shows mixed and conflicting findings, we observe that web-based tools may be useful in improving mental health outcomes, positively influencing treatment-seeking intentions, and actual treatment-seeking behavior for depression among postpartum adolescents [42,47]. Conversely, readily available information on the internet may increase anxiety among adolescent recipients [43]. The difference appears to lie in the source of information, whether curated by health care professionals specifically to address common concerns of the target group and presented in a controlled setting or self-acquired data sourced from various websites initiated by the adolescent recipients themselves. RCTs using similar platforms, not limited to adolescent mothers, yield similar results, with postpartum mothers with depressive symptoms describing significantly improved parenting competence and decreased depression severity following social media interventions compared to unexposed mothers [48-51]. The presence of a professionally moderated program may be a key differentiating factor in mental health outcomes. Web-based tools may also be more influential on mental health outcomes due to the users’ relative anonymity compared to conventional care. A systematic review found that although online services did not significantly facilitate mental help-seeking behavior in youths, many youths regularly use online services and recommend them to peers as they are easily accessible, anonymous, and less stigmatizing [52].

Aside from mental health outcomes, there may be other benefits for adolescent mothers, including fewer emergency room visits (with increased knowledge and confidence in postpartum care) [41] and positive, albeit limited, dietary and behavioral changes [44]. Vulnerable populations like low-income adolescent mothers do not have ready access to health care professionals or services, but frequently have questions regarding childcare. These require rapid attention and resolution, resulting in unnecessary emergency room visits. Despite a lack of theoretical knowledge, structured interventions can motivate both adolescents and adults to seek healthier lifestyles. Furthermore, professional support via web-based tools has demonstrated positive impact on both infants [53,54] and older mothers [55-57] in the general prenatal population.

Preexisting social media platforms and internet-based tools play an integral role in an adolescent’s life and are associated with positive and negative outcomes, particularly with maintaining beneficial friendships and fostering precarious and potentially dangerous relationships, respectively [45,46]. Despite some negative consequences, Sherman et al [32] found online adolescent pregnancy communities such as online support groups and discussion forums to be largely supportive and to serve an important role for those who use them. The authors noted that adolescents should choose their online community carefully to reduce additional psychological distress. Social media platforms may thus fill an unmet need to engage adolescent pregnant women, given the substantial use of social media globally [58].

Only 2 studies compared the efficacy of web-based tools and conventional methods in influencing maternal and neonatal outcomes in the adolescent population [41,42]. Mental health was the only common outcome that both studies evaluated, in which Logsdon et al [42] had a greater focus on depression compared to Hudson et al [41]. As such, no meaningful conclusions can be drawn to address our second research question.

A Singaporean study described increased risks of perinatal complications like anemia and preterm births and reduced likelihood of regular clinic attendance and sexually transmitted infection screening in a group of younger, vulnerable, predominantly Malay parturients with poorer access to prenatal care [59]. This highlights the importance of increasing accessibility to prenatal care and making a concerted effort to improve outreach [59]. Such young women are vulnerable to poor health literacy [60], closely related to eHealth literacy, which involves the use of digital technology [61], and are less likely to gain positive outcomes from internet searches [62]. eHealth interventions have proven effective in reaching out to populations with low levels of literacy [63], enabling health care professionals to facilitate behavioral change, personalize management, and improve education surrounding their health [64]. Aside from designing interventions to reach out to adolescents, it is also important to educate them and fill in the skill gap in eHealth literacy so they can access and evaluate health information accurately [65]. The use of professionally curated web-based tools incorporating online resources, communication services with health care professionals and peers, and social support may hold the key to reduce this important health inequality gap. However, larger age and culturally appropriate RCTs are necessary to validate the efficacy of these nontraditional methods.

Overall, our findings demonstrate a paucity of studies in this important aspect of managing adolescent pregnancies and highlight the effectiveness of web-based tools to reach pregnant and postpartum adolescents who may be more comfortable seeking help on online platforms. These tools allow health care professionals and policy makers to spread valuable pregnancy-related information to this vulnerable population in an age and peer acceptable way. Local or regional governments can potentially harness internet platforms and social media to drive public health policies. It may prove to be a more efficient allocation of resources, improving compliance to prenatal follow-ups and reducing pregnancy-related complications. Aside from antenatal care, governments can broaden their focus and cover topics pertaining to general women’s health and, importantly, contraception use.

### Strengths and Limitations

Our systematic review is timely as advanced technology and social media use are pervasive in modern societies, particularly among youth. Including both quantitative and qualitative studies in the review allowed for a broader interpretation of both statistical outcomes and adolescents’ qualitative feedback.

Key limitations of this systematic review are the weaknesses inherent in the included studies and the lack of research in this important area, particularly in Asian societies, despite being the most connected globally. Notwithstanding the extensive internet and social media use among adolescents, few studies have been conducted to directly establish the relationship between technology use in pregnant adolescents or adolescent mothers and pregnancy outcomes. Across the 7 studies there was great variability in measurement scales and reported outcomes, contributing to the heterogeneity of our results, and making it challenging to draw well-grounded conclusions. For this reason, we were not able to conduct a meta-analysis and were only able to provide a descriptive narrative of the studies. These studies were based in the United States and Western Australia, and findings may not be directly applicable to more ethnically diverse populations given the cultural differences in family traditions, community infrastructure, and child-raising practices [66]. Babes Pregnancy, a charity based in Singapore [67], has used social media to support these vulnerable youth, although the impact of this program is not known.

### Conclusion

The vastness of the information available on the web limits the ability of health care professionals to monitor or control sources of information sought by patients. It is important to create professionally curated platforms that patients can be directed to in order to prevent or limit exposure to potentially misleading or inaccurate information. Although our study is limited by the scope of the included studies, it is evident that web-based tools have the potential to improve outcomes in adolescent pregnancies. This review highlights the potential for web-based tools to target this vulnerable population, which is usually excluded from public health policies. The data presented can be highly informative to local health care policy makers. Overall, community-specific research is urgently needed to explore the potential of social media to guide novel interventions for this important vulnerable group.

## Appendix

Multimedia Appendix 1 Search strategy.

Multimedia Appendix 2 Risk of bias.

## Abbreviations

PRISMA: Preferred Reporting Items for Systematic Reviews and Meta-analyses
PROSPERO: International Prospective Register of Systematic Reviews
RCT: randomized controlled trial
