# Supplementary material for: The Influence of Web-Based Tools on Maternal and Neonatal Outcomes in Pregnant Adolescents or Adolescent Mothers: Mixed Methods Systematic Review
Source: J Med Internet Res. 2021 Aug 26;23(8):e26786. doi: 10.2196/26786 (PMC8430830; doi:10.2196/26786)
Supplement: Multimedia Appendix 1 [file jmir_v23i8e26786_app1.doc]

PubMed Search Strategy

(("pregnancy in adolescence"[MeSH Terms] OR ("pregnancy"[MeSH Terms] AND "adolescent"[MeSH Terms])) OR ((((((("adolescen*"[Title/Abstract] OR "teen*"[Title/Abstract]) OR "young"[Title/Abstract]) OR "youth"[Title/Abstract]) OR "high school*"[Title/Abstract]) OR "minor*"[Title/Abstract]) OR "girl*"[Title/Abstract]) AND ((("pregnan*"[Title/Abstract] OR "mother*"[Title/Abstract]) OR "birth"[Title/Abstract]) OR "maternal"[Title/Abstract]))) AND ("communications media"[MeSH Terms] OR (((((((((((((((((("social media"[Title/Abstract] OR "social network*"[Title/Abstract]) OR "social network site*"[Title/Abstract]) OR "forum*"[Title/Abstract]) OR "chatroom*"[Title/Abstract]) OR "message board*"[Title/Abstract]) OR "digital media"[Title/Abstract]) OR "new media"[Title/Abstract]) OR "new digital media"[Title/Abstract]) OR "internet"[Title/Abstract]) OR "website*"[Title/Abstract]) OR "technology"[Title/Abstract]) OR "telehealth"[Title/Abstract]) OR "e-health"[Title/Abstract]) OR "m-health"[Title/Abstract]) OR "interpersonal communication*"[Title/Abstract]) OR "mass communication*"[Title/Abstract]) OR "patient-patient"[Title/Abstract]) OR "patient to patient"[Title/Abstract]))

Scopus Search Strategy

( TITLE-ABS-KEY ( ( adolescen*  OR  teen*  OR  young  OR  youth  OR  "high school*"  OR  minor*  OR  girl* )  W/3  ( pregnan*  OR  mother*  OR  birth  OR  maternal ) ) )  AND  ( TITLE-ABS-KEY ( "social media"  OR  "social network*"  OR  "social network* site*"  OR  forum*  OR  chatroom*  OR  "message board*"  OR  "digital media"  OR  "new media"  OR  "new digital media"  OR  internet  OR  website*  OR  technology  OR  telehealth  OR  e-health  OR  m-health  OR  "interpersonal communication*"  OR  "mass communication*"  OR  "patient-patient"  OR  "patient to patient" ) )

CINAHL Plus Search Strategy

( ( ( (MH "Adolescence+") AND (MH "Pregnancy+") ) OR (MH "Pregnancy in Adolescence+") ) OR ( TI ( (adolescen* OR teen* OR young OR youth OR "high school*" OR minor* OR girl*) w3 (pregnan* OR mother* OR birth OR maternal) ) OR AB ( (adolescen* OR teen* OR young OR youth OR "high school*" OR minor* OR girl*) w3 (pregnan* OR mother* OR birth OR maternal) ) ) ) AND ( (MH "Communications Media+") OR ( TI ( "social media" OR "social network*" OR "social network* site*" OR forum* OR chatroom* OR "message board*" OR "digital media" OR "new media" OR "new digital media" OR internet OR website* OR technology OR telehealth OR e-health OR m-health OR "interpersonal communication*" OR "mass communication*" OR "patient-patient" OR "patient to patient" ) OR AB ( "social media" OR "social network*" OR "social network* site*" OR forum* OR chatroom* OR "message board*" OR "digital media" OR "new media" OR "new digital media" OR internet OR website* OR technology OR telehealth OR e-health OR m-health OR "interpersonal communication*" OR "mass communication*" OR "patient-patient" OR "patient to patient" ) ) )

PsychINFO Search Strategy

(exp Adolescent Mothers/ or exp Adolescent Pregnancy/ or ((adolescen* or teen* or young or youth or "high school*" or minor* or girl*) adj3 (pregnan* or mother* or birth or maternal)).ti,ab.) and (exp Communications Media/ or ("social media" or "social network*" or "social network* site*" or forum* or chatroom* or "message board*" or "digital media" or "new media" or "new digital media" or internet or website* or technology or telehealth or e-health or m-health or "interpersonal communication*" or "mass communication*" or "patient-patient" or "patient to patient").ti,ab.)
