# Supplementary material for: The Influence of Web-Based Tools on Maternal and Neonatal Outcomes in Pregnant Adolescents or Adolescent Mothers: Mixed Methods Systematic Review
Source: J Med Internet Res. 2021 Aug 26;23(8):e26786. doi: 10.2196/26786 (PMC8430830; doi:10.2196/26786)
Supplement: Multimedia Appendix 2 [file jmir_v23i8e26786_app2.doc]

Risk of Bias: Quality assessment

**Qualitative studies**

Assessed via the Joanna Briggs Institute (JBI) instrument

1. Is there congruity between the stated philosophical perspective and the research methodology
2. Is there congruity between the research methodology and the research question or objectives?
3. Is there congruity between the research methodology and the methods used to collect data?
4. Is there congruity between the research methodology and the representation and analysis of data?
5. Is there congruity between the research methodology and the interpretation of results?
6. Is there a statement locating the researcher culturally or theoretically?
7. Is the influence of the researcher on the research, and vice- versa, addressed?
8. Are participants, and their voices, adequately represented?
9. Is the research ethical according to current criteria or, for recent studies, and is there evidence of ethical approval by an appropriate body?
10. Do the conclusions drawn in the research report flow from the analysis, or interpretation, of the data?

Legend:

Y=Yes, N=No, U=Unclear, NA=Not applicable

Rating: I=Include, E=Exclude, S=Seek further info

| Authors | 1 | 2 | 3 | 4 | 5 | 6 | 7 | 8 | 9 | 10 | Rating |
| --- | --- | --- | --- | --- | --- | --- | --- | --- | --- | --- | --- |
| Fleming et al. | Y | Y | Y | Y | Y | Y | U | Y | Y | Y | I |
| Nolan et al. | Y | Y | Y | Y | Y | Y | Y | Y | Y | Y | I |
| Rueda et al. | Y | Y | Y | Y | Y | Y | Y | Y | Y | Y | I |

**Non-randomized controlled trials**

Assessed via the JBI instrument

1. Is it clear in the study what is the ‘cause’ and what is the ‘effect’ (i.e. there is no confusion about which variable comes first)?
2. Were the participants included in any comparisons similar?
3. Were the participants included in any comparisons receiving similar treatment/care, other than the exposure or intervention of interest?
4. Was there a control group?
5. Were there multiple measurements of the outcome both pre and post the intervention/exposure?
6. Was follow up complete and if not, were differences between groups in terms of their follow up adequately described and analyzed?
7. Were the outcomes of participants included in any comparisons measured in the same way?
8. Were outcomes measured in a reliable way?
9. Was appropriate statistical analysis used?

Legend:

Y=Yes, N=No, U=Unclear, NA=Not applicable

Rating: I=Include, E=Exclude, S=Seek further info

| Authors | 1 | 2 | 3 | 4 | 5 | 6 | 7 | 8 | 9 | Rating |
| --- | --- | --- | --- | --- | --- | --- | --- | --- | --- | --- |
| Logsdon et al. (2013) | Y | NA | NA | N | Y | NA | NA | Y | U | I |

**Cohort studies**

Assessed via the Newcastle-Ottawa Quality Assessment Scale (NOS)

Selection

1) Representativeness of the exposed cohort
a) truly representative of the average _______________ (describe) in the community *

b) somewhat representative of the average ______________ in the community *
c) selected group of users eg nurses, volunteers
d) no description of the derivation of the cohort

2) Selection of the non exposed cohort
a) drawn from the same community as the exposed cohort *

b) drawn from a different source
c) no description of the derivation of the non exposed cohort

3) Ascertainment of exposure
a) secure record (eg surgical records) *

b) structured interview *
c) written self report
d) no description

4) Demonstration that outcome of interest was not present at start of study

a) yes *

b) no

Comparability

1) Comparability of cohorts on the basis of the design or analysis
a) study controls for _____________ (select the most important factor) *
b) study controls for any additional factor * (This criteria could be modified to indicate specific control for a second important factor.)

Outcome

1) Assessment of outcome
a) independent blind assessment *

b) record linkage *
c) self report
d) no description

2) Was follow-up long enough for outcomes to occur
a) yes (select an adequate follow up period for outcome of interest) *

b) no

3) Adequacy of follow up of cohorts
a) complete follow up - all subjects accounted for *
b) subjects lost to follow up unlikely to introduce bias - small number lost - > ____ % (select an

adequate %) follow up, or description provided of those lost) *

c) follow up rate < ____% (select an adequate %) and no description of those lost

d) no statement

Legend:

S=Selection; C=Comparability; O=Outcome

| Author | S1 | S2 | S3 | S4 | C1 | O1 | O2 | O3 | Stars awarded | | |
| --- | --- | --- | --- | --- | --- | --- | --- | --- | --- | --- | --- |
|  |  |  |  |  |  |  |  |  | S | C | O |
| Logsdon et al. (2018) | b(*) | a(*) | a(*) | b | a(*), b(*); race, socioeconomic status, number of adolescent births | c | a(*) | b(*); (2.1% in control group, 3.1% in intervention group) | *** | ** | ** |

**Randomized controlled trials**

Assessed via the Cochrane Risk of Bias 2 (RoB 2)

1. Was the allocation sequence random?
2. Was the allocation sequence concealed until participants were enrolled and assigned to interventions?
3. Did baseline differences between intervention groups suggest a problem with the randomization process?
4. Risk-of-bias judgement.
5. Were participants aware of their assigned intervention during the trial?
6. Were carers and people delivering the interventions aware of participants' assigned intervention during the trial?
7. If Y/PY/NI to 2.1 or 2.2: Were there deviations from the intended intervention that arose because of the trial context?
8. If Y/PY to 2.3: Were these deviations likely to have affected the outcome?
9. If Y/PY/NI to 2.4: Were these deviations from intended intervention balanced between groups?
10. Was an appropriate analysis used to estimate the effect of assignment to intervention?
11. If N/PN/NI to 2.6: Was there potential for a substantial impact (on the result) of the failure to analyse participants in the group to which they were randomized?
12. Risk-of-bias judgement.
13. Were data for this outcome available for all, or nearly all, participants randomized?
14. If N/PN/NI to 3.1: Is there evidence that the result was not biased by missing outcome data?
15. If N/PN to 3.2: Could missingness in the outcome depend on its true value?
16. If Y/PY/NI to 3.3: Is it likely that missingness in the outcome depended on its true value?
17. Risk-of-bias judgement.
18. Was the method of measuring the outcome inappropriate?
19. Could measurement or ascertainment of the outcome have differed between intervention groups?
20. If N/PN/NI to 4.1 and 4.2: Were outcome assessors aware of the intervention received by study participants?
21. If Y/PY/NI to 4.3: Could assessment of the outcome have been influenced by knowledge of intervention received?
22. If Y/PY/NI to 4.4: Is it likely that assessment of the outcome was influenced by knowledge of intervention received?
23. Risk-of-bias judgement.
24. Were the data that produced this result analysed in accordance with a pre-specified analysis plan that was finalized before unblinded outcome data were available for analysis?
25. Is the numerical result being assessed likely to have been selected, on the basis of the results, from multiple eligible outcome measurements (e.g. scales, definitions, time points) within the outcome domain?
26. Is the numerical result being assessed likely to have been selected, on the basis of the results, from multiple eligible analyses of the data?
27. Risk-of-bias judgement.
28. Overall. Risk-of-bias judgement.

Legend:

Y=Yes, PY=Probably yes, PN=Probably no, N=No, NI=No information, NA=Not applicable

Rating; L=Low, H=High, SC=Some concerns

| Author | 1.1 | 1.2 | 1.3 | 1.4 | 2.1 | 2.2 | 2.3 | 2.4 | 2.5 | 2.6 | 2.7 | 2.8 | 3.1 | 3.2 |
| --- | --- | --- | --- | --- | --- | --- | --- | --- | --- | --- | --- | --- | --- | --- |
| Hudson et al. | Y | Y | N | L | Y | Y | N | NA | NA | Y | NA | L | Y | NA |

| Author | 3.3 | 3.4 | 3.5 | 4.1 | 4.2 | 4.3 | 4.4 | 4.5 | 4.6 | 5.1 | 5.2 | 5.3 | 5.4 | 6 |
| --- | --- | --- | --- | --- | --- | --- | --- | --- | --- | --- | --- | --- | --- | --- |
| Hudson et al. | NA | NA | L | N | N | NI | PY | PN | L | Y | Y | PN | L | L |

**Mixed methods studies**

Assessed via Mixed Methods Appraisal Tool

Screening Questions

S1. Are there clear qualitative and quantitative research questions (or objectives*), or a clear mixed methods question (or objective*)?

S2. Do the collected data allow address the research question (objective)? E.g., consider whether the follow-up period is long enough for the outcome to occur (for longitudinal studies or study components)

Questions

1. Are the sources of qualitative data (archives, documents, informants, observations) relevant to address the research question (objective)?
2. Is the process for analyzing qualitative data relevant to address the research question (objective)?
3. Is appropriate consideration given to how findings relate to the context, e.g., the setting, in which the data were collected?
4. Is appropriate consideration given to how findings relate to researchers’ influence, e.g., through their interactions with participants?
5. Is there a clear description of the randomization (or an appropriate sequence generation)?
6. Is there a clear description of the allocation concealment (or blinding when applicable)?
7. Are there complete outcome data (80% or above)?
8. Is there low withdrawal/drop-out (below 20%)?
9. Are participants (organizations) recruited in a way that minimizes selection bias?
10. Are measurements appropriate (clear origin, or validity known, or standard instrument; and absence of contamination between groups when appropriate) regarding the exposure/intervention and outcomes?
11. In the groups being compared (exposed vs. non-exposed; with intervention vs. without; cases vs. controls), are the participants comparable, or do researchers take into account (control for) the difference between these groups?
12. Are there complete outcome data (80% or above), and, when applicable, an acceptable response rate (60% or above), or an acceptable follow-up rate for cohort studies (depending on the duration of follow-up)?
13. Is the sampling strategy relevant to address the quantitative research question (quantitative aspect of the mixed methods question)?
14. Is the sample representative of the population understudy?
15. Are measurements appropriate (clear origin, or validity known, or standard instrument)?
16. Is there an acceptable response rate (60% or above)?
17. Is the mixed methods research design relevant to address the qualitative and quantitative research questions (or objectives), or the qualitative and quantitative aspects of the mixed methods question (or objective)?
18. Is the integration of qualitative and quantitative data (or results*) relevant to address the research question (objective)?
19. Is appropriate consideration given to the limitations associated with this integration, e.g., the divergence of qualitative and quantitative data (or results*) in a triangulation design?

Legend:

Y=Yes, N=No, C=Can't tell

| Author | S1 | S2 | 1.1 | 1.2 | 1.3 | 1.4 | 2.1 | 2.2 | 2.3 | 2.4 | 3.1 |
| --- | --- | --- | --- | --- | --- | --- | --- | --- | --- | --- | --- |
| Vander Wyst et al. | Y | Y | Y | Y | Y | C | NA | NA | NA | NA | NA |

| Author | 3.2 | 3.3 | 3.4 | 4.1 | 4.2 | 4.3 | 4.4 | 5.1 | 5.2 | 5.3 |
| --- | --- | --- | --- | --- | --- | --- | --- | --- | --- | --- |
| Vander Wyst et al. | NA | NA | NA | C | C | Y | Y | Y | Y | N |
